# Wearable devices and ecological momentary assessment EMA in the workplace: A study protocol on work stress assessment

**DOI:** 10.1371/journal.pone.0339288

**Published:** 2026-01-23

**Authors:** Giuseppina Dell’Aversana, Margherita Herold, Silvia Simbula, Isabella Ruina, Margherita Pillan, Massimo Miglioretti

**Affiliations:** 1 Department of Psychology, University of Milano-Bicocca, Piazza dell’Ateneo Nuovo, Milan, Italy; 2 Bicocca Center for Applied Psychology, University of Milano-Bicocca, Piazza dell’Ateneo Nuovo, Milan, Italy; 3 Department of Design, Politecnico di Milano; Helwan University Faculty of Engineering, EGYPT

## Abstract

Work-related stress (WRS) remains a significant concern in occupational health. Despite its significance, measuring WRS presents methodological challenges. Advancements in real-time data collection methods offer new opportunities to enhance the accuracy of WRS assessment. This study proposes an innovative, participatory protocol for assessing WRS. The approach integrates subjective self-reported measures, collected through ecological momentary assessment (EMA), and physiological monitoring via wearable smartwatches. By combining time-contingent and event-contingent sampling, the methodology enables continuous tracking of stress responses throughout the workday, providing a more dynamic and context-sensitive understanding of workplace stress. A key objective of this research is to evaluate the feasibility and acceptability of implementing this protocol in real-world organizational settings. By incorporating participatory design principles, the methodology actively involves workers in the assessment process, ensuring that the tools and procedures are both effective and user-friendly. This participatory approach fosters engagement and compliance, ultimately improving the quality of collected data. This study contributes to developing more robust and ecologically valid stress assessment methods. Integrating real-time monitoring with self-reported data represents a promising direction for occupational health research, paving the way for more targeted and evidence-based interventions to mitigate workplace stress.

## Introduction

There is a pressing need to assess the psychosocial aspects of the workplace and enhance employee well-being. Work-related stress (WRS) stands out as one of the most critical concerns in occupational health management [1,2]. Organizational psychology literature has traditionally conceived stress as a psychological state that reflects a broader interaction process between the individual and their work environment [3]. Stress is defined as the emotional, cognitive, behavioral, and physiological reaction to aversive and noxious aspects of work, work environments, and work organizations. It is a state characterized by high arousal levels, often accompanied by feelings of not coping [4]. It is well known that WRS negatively impacts both workers and organizations. Work-related stress has been linked to several health issues, including cardiovascular disease [5], musculoskeletal disorders such as back problems [6], and repetitive strain injuries affecting the neck, shoulders, arms, wrists, and hands [7]. Additionally, it is associated with increased absenteeism (e.g., [8,9]) and turnover [10]. Work-related stress is a major cost for companies and countries, reducing productivity through absenteeism and presenteeism (working while unwell). Despite efforts to prevent it, stress remains a significant issue for occupational health, worsened by the pandemic. Managing psychosocial risks has become increasingly complex due to technological advancements, the rise of remote and hybrid work arrangements, changes in employment contracts, and the growing diversity of the workforce [11]. The OSH Pulse survey [12] revealed a relatively high level of self-reported work-related stress and mental health issues. Specifically, 26.8% of respondents reported stress, depression, or anxiety caused or exacerbated by work, and 44% indicated that their work stress had increased due to the pandemic. A high workload is one of the main factors linked to stress, but the survey highlights that the lack of organizational measures to mitigate it is another key factor affecting workers’ health. Supporting organizations in assessing and managing stress remains a key objective [12]. In recent decades, following the EU framework Directive 1989/391/EEC on occupational safety and health, European countries established policies to address stress-related issues, requiring companies to assess work-related stress risks (WRS) and implement actions to prevent, reduce, and eliminate potential stressors. The control cycle approach [13] was proposed to address this issue. It provides a systematic problem-solving framework to improve work-related stress (WRS) continuously. Typically, assessing WRS involves various activities, including consultations with employees. Since stress is recognized as a psychological state, its measurement has traditionally relied on self-report measures focusing on the appraisal process and emotional experiences associated with stress. According to the established framework, WRS assessments necessitate collecting data regarding job content and contextual factors through interviews, questionnaires, and focus groups within organizations. This process is essential for identifying hazards, determining those at risk, and evaluating and prioritizing the associated risks (see the Italian context). [14]). Following the assessment, a management phase involves implementing improvements to prevent and reduce stress, as well as monitoring workers’ health. The design of the assessment, along with the instruments and procedures used, is crucial to ensuring the quality of the data collected. However, the literature has ongoing debate regarding ensuring data quality and improving working conditions [15]. Methodologically, much attention has been paid, on the one hand, to mechanisms of worker participation and, on the other, to the use of triangulation. The triangulation principle holds that a potential psychosocial or organizational hazard must be identified by cross-referencing different types of evidence. Recently, there has been growing interest in using day-level measurement to assess day-to-day stress. To this end, EMA and wearable devices have been used to collect the daily stress experiences and to track physiological stress responses [16]. These methodologies could be promisingly employed for assessing stress in small and medium-sized enterprises for limited numbers of at-risk workers [17].

This research addresses the need to develop new methodologies for assessing work-related stress, focusing on office work in the Italian context. This study was part of the MUSA – Multilayered Urban Sustainability Action research ecosystem, funded by the Ministry of University and Research under the National Recovery and Resilience Plans, addressing the Spoke 2 challenge of Big Data in Life Sciences. Research goals align with MUSA’s Work Packages (WP): WP3 focuses on improving global health with big data, while WP4 develops customizable tools for continuous monitoring and wellbeing. The protocol results from collaboration between the BICApP laboratory (work psychology) and the IEX-Interaction and Experience Design Lab (user-centered design). This interdisciplinary approach promotes participatory design, encouraging workers to actively engage in the development of protocols for assessing work-related stress [18]. This document proposes a methodology for assessing work-related stress through a participatory model. This model, based on the principle of triangulation, integrates subjective measures—including workers’ responses from questionnaires and interviews—experience sampling methods (EMA), which consist of brief surveys conducted during the workday to capture real-time emotional states or stress levels, and physiological measures such as heart rate variability. The protocol outlined in this document also seeks to evaluate the acceptability of the entire work-related stress assessment process while gathering daily data on workplace stressors and their psychological effects. Specifically, it assesses the feasibility of implementing the protocol and measures employees’ willingness to engage with it. The research questions guiding this study are the follows.

The first research question is: *“What benefits can integrating subjective measures—both individual and collective—with objective or physiological indicators offer in assessing and managing work-related stress?”* This study will collect data on perceived work-related stress and well-being using focus groups, EMA, and Fitbit monitoring. Expected outcomes include the identification of contextual stressors and resources, as well as perceived stress, which are factors that enhance workers’ acceptability of the methods and instruments in the protocol.

The second research question is: *“What critical challenges might arise in employing such an approach, considering practical, ethical, managerial, and employee-related aspects?”* Expected outcomes focus on evidence on methodological limitations in the integration of data, on technical challenges related to device use and data collection and management; factors that reduce workers’ acceptability of methods and instruments in the protocol, providing a critical reflection on the usefulness of the data for management decisions.

### Theoretical background

#### Definition of work-related stress.

Successfully managing stress in the workplace requires a shared understanding of the definition of stress. Our theoretical framework adopts a psychological approach, emphasizing the influence of environmental, psychosocial, and organizational factors on work-related stress. Stress results from problematic interactions between individuals and their environments, shaped by cognitive processes and emotional reactions. It is a state characterized by high levels of arousal and distress, often by feelings of not coping. Key work-related stressors can be operationalized through two main job characteristics: job demands and job resources. These elements are central to several work stress models [e.g., [19–21]. Stress reactions may manifest emotionally, cognitively, behaviorally, or physiologically when individuals are exposed to workplace risk factors. When personal resources are strained, this can trigger a range of possible reactions, including physiological responses like increased heart rate, elevated blood pressure, and hyperventilation; emotional responses, such as feelings of nervousness or irritation; cognitive effects, including reduced attention, impaired perception, and forgetfulness; and behavioral reactions, such as impulsive actions and an increased likelihood of making mistakes [3]. Our study and this research protocol were developed within the theoretical framework of the Job Demands-Resources (JD-R), recognizing the multidimensional nature of work-related stress. In particular, JD-R theory frames job stress as an imbalance between job demands and resources. Job demands, including physical, psychological, social, and organizational factors, require sustained effort and are negatively related to employee well-being [22,23]. In contrast, job resources play a motivational role and help mitigate the adverse effects of job demands, encompassing social, organizational, and developmental aspects. The JD-R model is widely used to assess occupational health, offering a flexible framework that considers both negative and positive aspects of work. It allows for the inclusion of all relevant job characteristics and can be tailored to the specific needs of any organization [24]. The model also recognizes that individual factors—such as personality, values, age, gender, education, and family situation—affect one’s ability to cope with stress.

#### Multi-method approaches.

Stress measurement, however, poses significant methodological challenges. Traditionally, most data on stress comes from subjective self-report tools, such as questionnaires and scales that rely on workers’ perceptions of their working conditions [25]. Self-reports are used to measure workers’ perceptions of sources of stress and workplace resources and assess work health outcomes such as perceived stress and strain, as well as engagement. While valuable for understanding worker experiences, they can be biased due to emotional states or intentional distortion[26]. Additionally, the issue of “negative affectivity” (NA) can distort perceptions of both the work environment and well-being [27]. The reliance on self-reports for stressors and reactions also introduces common method variance [28]. Objective measures, such as observations and archival data, reduce some biases but come with their limitations. For instance, absenteeism and performance records may not fully reflect psychosocial risks due to other influencing factors [29]. Researchers increasingly adopt multi-method approaches that combine subjective, objective, qualitative, and quantitative measures to address these challenges. Following the triangulation principle [29], integrating different methods provides convergence and corroboration, where results from one method can clarify or complement findings from another. This research protocol focuses on complementarity, where results from one method enhance the understanding of another, as well as development, where insights from one method inform subsequent research design. We aim to test the combined use of qualitative and quantitative methods by collecting both subjective data (workers’ perceptions of work stress) and objective data (physiological data) from various sources, including focus groups, EMA, and wearable devices.

#### Ecological momentary assessment (EMA).

Methodologically, current trends suggest moving beyond cross-sectional studies to incorporate time as a variable, integrate multiple data sources, and consider the organizational context. Collecting psychological data at a single point presents significant limitations when attempting to capture exposure to stressors that may fluctuate unpredictably throughout the day. Furthermore, these single-timepoint assessments are susceptible to recall bias [30]. EMA has shown promise in capturing inter-individual differences and intra-individual changes in job satisfaction and stress responses [31]. EMA involves repeated data collection of participant behaviors and experiences in real-time in the natural environment [32]. Data is increasingly collected multiple times over a set period through digital platforms like smartphone apps. Recent research has shown that EMA is a viable method for studying work stress. EMA offers several advantages over traditional epidemiological approaches, particularly by capturing individual differences and fluctuations in daily experiences [15]. Lukan and colleagues [33] analysed existing research that explores day-to-day stressors, highlighting that only a few studies measured physiological outcomes. Recent research studied daily stress using the Straw Protocol. Over 15 days, participants answered questionnaires every 90 minutes through an app, gathering data on job demands, resources, health outcomes, and coping strategies. Similarly, Weale and colleagues [30] proposed a protocol that collected data on contextual stress factors and health outcomes but additionally considered musculoskeletal pain outcomes. An important consideration is obtaining reliable and repeated data without overburdening participants [30,34]. Talbot [34] found low acceptability and moderate feasibility of the STRAW protocol in Australia, with only 49.1% completing the questionnaire due to the burden of frequent assessments. Weale and colleagues [30] addressed this by limiting the data collection to 10 days and three EMAs: morning, daytime, and evening. A follow-up study by the same authors [35] found that specific recommendations should be implemented, while the protocol was generally feasible and well-accepted. For instance, the study used the Empatica E4 (a medical-grade wearable device in the form of a wristband that offers real-time physiological data acquisition and streaming) to collect physiological indicators such as heart rate, skin conductance, and temperature. These measures were relevant for capturing objective stress markers in everyday work contexts. However, some participants described the device as “clunky” and “uncomfortable.” Consequently, the authors suggested adopting an alternative instrument for physiological data collection. Lukan et al. [33] reported that, although the EMAs were not perceived as overburdening, “most negative remarks [..] were about participants needing to get used to them and that they were sometimes difficult to answer on a 90-minute basis”. Based on these previous studies, we propose a protocol with the timing and frequency of questionnaires that will be amended. Also, we will include an event-contingent questionnaire during working hours, focusing on stressful events and their affective and behavioural responses with closed and open-ended questions.

#### Psychophysiological methods.

Physiological measures can complement self-reports by capturing physical arousal states associated with reactions to stressors and health outcomes or perceptions of strain. Psychophysiological methods provide discrete, continuous, and dynamic measures of stress, enabling researchers to assess emotional states or preferences that may occur before conscious awareness [36]. Incorporating psychophysiological data diversifies information sources, helping to reduce bias and leading to more robust and accurate assessments of work-related stress [17]. Physiological data add another layer of insight into stress measurement. Objective physiological data rely on theories recognizing the activation of the central and peripheral nervous systems and cardiovascular, neuroendocrine, immune, and metabolic systems in response to stress [37]. The most frequently used measures of physiological responses to work-related stress include heart rate variability, interbit interval levels, blood pressure, and biochemical markers such as uric acid, blood glucose, steroid hormones (e.g., cortisol, including salivary cortisol), blood cholesterol, and catecholamines (adrenaline and noradrenaline) [38]. Given the complexity of stress responses, isolating the impact of work-related stress through a single biological marker, such as hormone levels, is challenging. A more effective approach involves collecting data from multiple sources simultaneously, such as self-reports, observational data, and physiological markers, to achieve more comprehensive and reliable insights [17]. Despite these advantages, psychophysiological measures are still not widely used in organizational research. Due to the potential for investigating psycho-physiological work-related stress, integrating subjective data (subjective experience gathered with interviews, focus groups, and EMA) and objective data (physiological parameters) defines the protocol’s methodological strategy.

#### Wearable for assessing WRS.

A growing number of research studies provide evidence of the widespread use of wearable technology in the work environment for monitoring (i.e., collecting quantified self-data) and tracking (i.e., prevention programs based on data) [16]. In this framework, the occupational health field considers wearable devices valuable opportunities for conducting work-related stress assessments. Some of the main advantages are the possibility of recording biometric signals without disrupting participants’ activities and supporting real-life stress monitoring [39,40]. Wearable devices could also enhance workers’ health awareness. For example, these devices enable individuals to recognize and adjust potentially harmful behaviors [41]. Device functionalities allow the employees to monitor their behaviors, moods, and progress, helping them to enhance their self-recognition and awareness. At the same time, this can help people decide to pursue certain habits, abandon them, or reflect on occurrences. Nowadays, wearable technology can be more and more personalized for the specificity of workplace contexts, promoting employees’ self-awareness and consequent behavioral and productivity changes [42,43]. Some advantages led by wearable technologies can be summarized in the unnecessary asking directly to employees [44] and in the possibility of personalized action directly to the well-being within the workplace context [45] and predicting an adequate path for prevention programs [46]. Low-impact wearable devices (wristband type) should minimize interference with the participant’s work and personal activities. However, using these devices also presents significant challenges, including concerns about privacy, potential distractions, accuracy, resistance, and long-term impacts [47]. To identify factors that may hinder the acceptance of wearable devices, experimental protocols should include an evaluation of the device’s acceptability for work-related assessment in organizations.

#### Technology acceptability.

Research highlights that technology acceptance is vital for the success of wearable devices used in evaluating work-related stress and well-being [48]. Over the last 30 years, several theoretical models have been presented to analyse and explain the adoption and behaviours associated with the introduction of technologies. Specifically, the Technology Acceptance Model (TAM) [49] is the most widely used theoretical model for explaining technology adoption intentions, as it can be applied to a wide range of technologies [50,51], wearable devices included [52]. Three main dimensions make up the model, namely perceived usefulness (PU), perceived ease of use (PEOU), and intention to use (INT). TAM states that the intention to use a new technology depends mainly on the perceived usefulness (i.e., the degree to which the technology is perceived as helpful in achieving one’s goals) and the perceived ease of use (i.e., the degree to which the technology is perceived as easy to use) of the technology. Therefore, if users find a technology useful and easy to use, they are more likely to adopt it (i.e., the intention to use). In addition, some theoretical expansions have been created, e.g., TAM2 [53], TAM3 [54], UTAUT [50], UTAUT 2 [55], even if the core of these models has always remained the same. The Unified Theory of Acceptance and Use of Technology (UTAUT) explains how people adopt technology by considering factors like performance, effort, social influence, and support. UTAUT2 expands on this with additional consumer-related factors. Specifically, several scholars have used TAM or UTAUT as a base model and extended it with the external or contextual factors derived from different theories [56].

Generally speaking, user acceptance is influenced not only by individual factors, but also by organisational and social factors. Organisational support, including managerial support and integration into the workplace culture, significantly impacts wearable technology adoption [50,57]. In addition, it has been shown that active participation in the design and implementation of wearable device-based interventions can increase user engagement, thereby fostering their adoption; this, in turn, could increase the likelihood of effective use of such devices in daily life [58]. Research by Orji and Moffatt [59] indicates that involving users in co-designing health technologies enhances their sense of ownership and motivation. For instance, employees who personalize stress management wearables are more likely to use them regularly. This aligns with participatory design literature, which suggests that user engagement improves both acceptance and effectiveness of health interventions [60,61]. Thus, assessing the feasibility and acceptability of multimodal work-related stress assessments is essential. Previous studies have evaluated objective measures such as retention rates, questionnaire completion rates, and the amount of time participants wore the devices [33]. While Weale et al. [30] examined participant experiences through interviews, subjective perceptions of wearable acceptability based on the TAM model were not systematically gathered. Additionally, there remains a significant lack of evidence regarding the advantages and disadvantages of commercial wearables.

## Materials and methods

### Study design

This project is a prospective observational study that utilizes data collected through focus groups, wearable technology, and repeated questionnaire measures. The protocol will undergo pilot testing to assess the acceptability and feasibility of the methods.

The methodology consists of several stages designed to test the protocol for assessing work-related risks (as presented in the next paragraph). It is particularly suited for small work environments. The novelty of this approach lies in its emphasis on overall acceptability throughout all phases, in line with the principles of participatory design [18,60]. Specifically, participant engagement is a fundamental prerequisite for evaluating the acceptability of the experience, particularly regarding the instruments used for data collection, as well as the timing and modalities. In this context, adopting participatory design methodologies and co-design represents a promising strategy to address the complexities associated with collecting and utilizing personal data through wearable devices [61,62].

To minimize the perceived burden on participants, the following measures will be implemented:

- The monitoring period will be limited to 10 days [30].
- The number of time-based EMAs will be reduced to two per day (morning and evening), with extended time slots to accommodate work and personal schedules. Additionally, an event-related questionnaire will be introduced during the daytime to capture specific working conditions.
- Based on feedback from Lukan et al. [63] regarding the challenges participants faced in adapting to the monitoring routine, the first week of data collection—including EMA and wearable device use—will serve as a run-in phase to facilitate adaptation.
- Unlike previous studies that employed high-tech devices (e.g., Empatica), this protocol will utilize a commercial device, the Fitbit Charge 5, a wrist-worn wearable that tracks heart rate, physical activity, sleep patterns, and stress indicators, providing continuous, user-friendly physiological and behavioral data. This decision aligns with the recommendations of Weale and colleagues [30], who advocate for using lower-impact monitoring tools to enhance participant compliance. Moreover, the use of commercially available wearables enhances the potential for scalability and real-world applicability, given their lower cost, accessibility, and user familiarity.

### Main activities

#### Phase 1: Involvement of the steering committee and management of the assessment process.

The process begins with the establishment of a steering group and the commitment of organizational leadership to define and share the goals, methods, and actions of the Work-Related Stress (WRS) assessment and management pathway. This initial phase involves identifying the key roles required to provide contextual information, such as organizational performance indicators, development systems, and work processes, and to manage and coordinate the assessment activities effectively. The steering group comprises executives and decision-makers who can support researchers in interpreting the data and overseeing the implementation of the process. This group remains active throughout the entire process to ensure that the specific objectives are achieved at each stage. A crucial responsibility of the steering group is to ensure clear and consistent communication with employees, particularly those involved in the various phases of the assessment and management activities.

#### Phase 2: Data collection.

**Fig 1:**
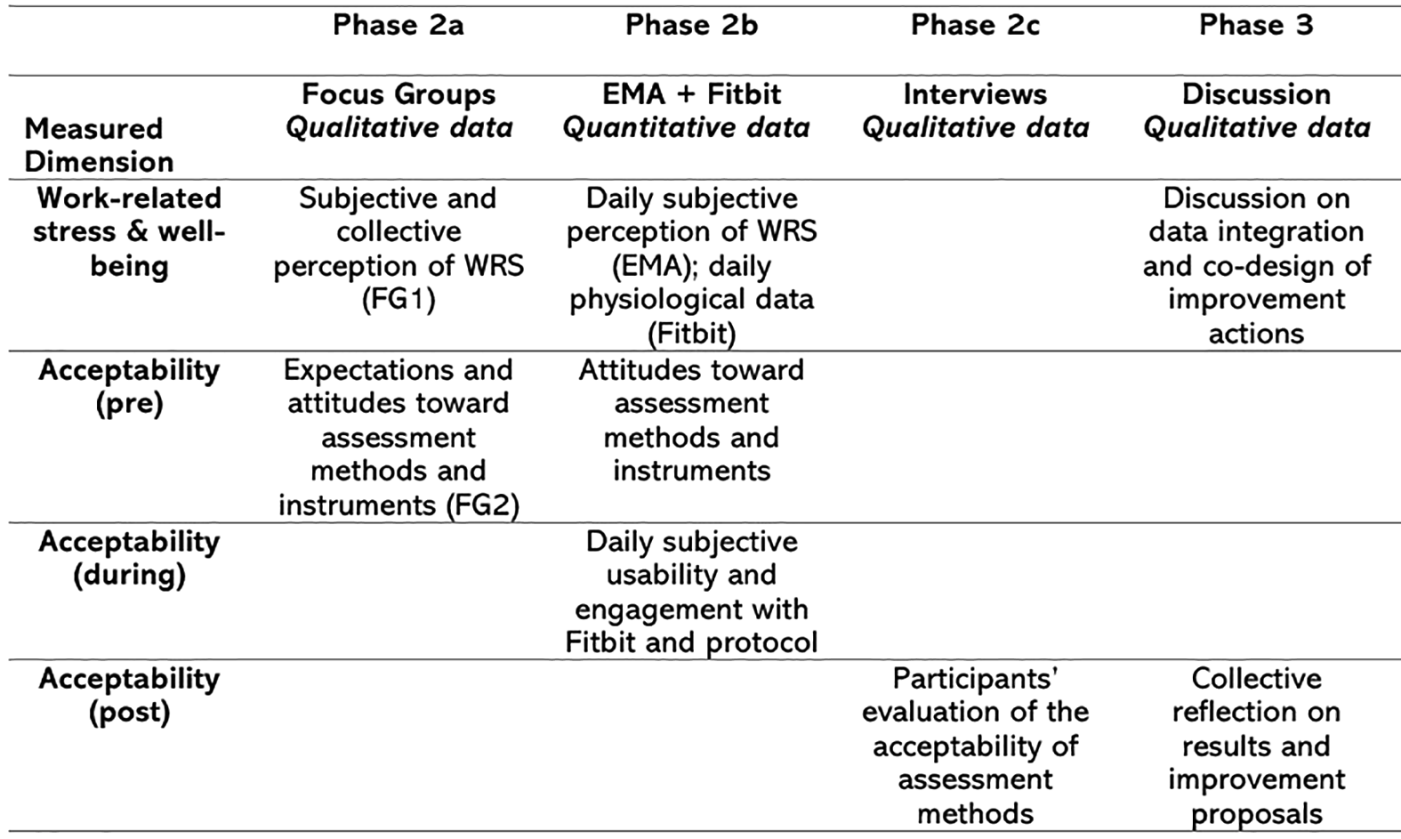
Mixed-methods design.

The second step consists of data collection, structured into three sub-stages. Following Creswell’s framework [64], the study employs a mixed-methods design that combines sequential and convergent elements. In the sequential component, data are gathered across three phases: an initial qualitative Phase 1 explores work-related stress and acceptability while also informing the design of subsequent EMA and wearable protocols; Phase 2 integrates qualitative and quantitative perspectives by comparing and contrasting findings; and Phase 3 extends this comparison for acceptability after the assessment. In the convergent component (Phase 2), subjective self-reports and objective physiological measures are collected simultaneously to examine their convergence and divergence. This design allows for both triangulation and complementarity, thereby providing a richer and more context-sensitive interpretation of workplace stress. Fig 1 provides a synthesis of our mixed-methods design.

#### 2a. Focus groups.

The first sub-stage involves the use of focus groups to explore participants’ perceptions of work stress in their work environment and their views on work-related stress assessment methods, as well as to examine job demands and resources within the specific work context. This stage also aims to assess participants’ expected acceptability of the assessment instruments and to gather information to design context-specific research tools. Two focus groups with the same participants are planned.

In the first session (FG1), participants will discuss both the positive and negative aspects of their work and organizational context using the Job Demands-Resources (J-DR) framework. They will reflect on the most common difficulties and available resources they have encountered in their work recently. This discussion aims to identify factors that are prevalent across different workers as well as those that vary by role. Participants will also address any variations or cyclical patterns in work-related stress and resources.

The goal is to provide insights that can guide decisions regarding the timing of data collection, support the interpretation of future data, facilitate comparisons between different data sources, and inform discussions at the end of the study to design targeted improvement actions. Additionally, this information could be used to incorporate extra measures in the Experience Sampling Method (EMA) to assess the impact of job stressors or resources on stress outcomes.

In the second session (FG2), participants will trial Fitbit and provide feedback on its perceived utility, facilitators, and barriers for assessing work-related stress. This focus group aims to collect data on participants’ acceptability prior to use and to gather data to refine methodological procedures for subsequent steps, such as EMA scheduling, smartphone compatibility, and tailored participant communication to optimize engagement in the following data collection. The focus group also serves as an opportunity for participants to become familiar with the research team, ask questions, and help reduce potential barriers or mistrust.

#### 2b. Ecological Momentary Assessment (EMA) and physiological data.

The second sub-stage envisages the use of EMA with Physiological Data. This aims to assess work-related stress by analysing day-to-day data on job demands and resources, health and well-being outcomes, coping strategies, perceived acceptability and physiological data. The study begins with a baseline questionnaire before collecting data through EMA and Fitbit. At baseline (T0), participants will complete a questionnaire assessing socio-demographics, workplace well-being, and the acceptability of wearable technology. Following this, data will be collected through EMA combined with physiological monitoring by Fitbit over ten working days (two weeks). The EMA is designed using an integrated approach of time-related and event-related data collection. During the first week (Monday to Friday, T1), participants will complete an evening questionnaire focused on the acceptability of the technology, using a time-related design. In the second week (Monday to Friday, T2), participants will complete three daily questionnaires. According to the time-related method, participants will respond to a morning questionnaire before starting their workday, assessing their well-being, and to an evening questionnaire after work, which will address both work-related stress outcomes and the acceptability of the Fitbit. Additionally, participants may complete an event-contingent questionnaire during working hours, focusing on stressful events and their affective and behavioural responses.

#### 2.c Individual Interviews about protocol acceptability.

The third sub-stage involves conducting individual interviews. After completing the data collection through EMA and wearables, semi-structured interviews will be held with all participants. This approach will allow for a deeper exploration of the data gathered regarding the adoption and acceptability of the wearable devices used to assess work-related stress and the research protocol.

#### Phase 3: Data analysis, discussion, proposals.

After collecting data in Phase 2, the information undergoes analysis and discussion. The insights and interpretations are presented to the steering committee for review, and then the results are shared with the respondents. Researchers also suggest potential interventions to address specific challenges within the context. Wherever possible, they initiate participatory activities to generate ideas and implement solutions.

### Setting and procedures

The protocol will be implemented in a working environment within academic institutions. The population is the administrative staff of university departments located in northern Italy. The inclusion criteria require the participant to agree to install the app on their smartphone and wear the Fitbit wristband continuously during working hours for 10 working days. We aimed to include 110 participants. Power analysis was carried out for the primary planned tests. For person-level correlations between physiological data (e.g., HR) and psychological measures (e.g., burnout), using G*Power (two-tailed, α = 0.05, power = 0.80), detecting a correlation of r = 0.30 requires approximately 84 participants. For the mixed-effects repeated measures design on job stress (5 measurements per participant, ICC = 0.38 according to previous research [15], power = 0.80), 97 participants are recommended to detect a small-to-moderate effect (β ≈ 0.20) of a single predictor on heart rate. This sample size accounts for the repeated measures’ structure and provides a buffer for potential participant drop-outs. Sensitivity analyses confirmed that this number can detect the expected effect size.

Initially, the research team will consult the head of the department and the administrative chief to present the methodology and objectives of the work-related stress risk assessment and obtain their approval for recruiting participants. Following this, a meeting will be organized with all technical-administrative staff members of the department to present the research project. After the meeting, an email will be sent to all staff, containing a Google Form to gauge their willingness to participate in Phase 2a, which involves focus groups. In addition to confirming their willingness to participate, the form will request socio-demographic information, which will help form the focus groups. The groups will be created based on internal heterogeneity criteria (e.g., age, seniority, gender), ensuring a balance of similarity between the two groups. To encourage participation and meet practical considerations, each focus group will consist of five participants [65]. Each session is expected to last approximately two hours and will be scheduled during working hours. The first session will focus on discussing well-being, work-related stress, and the challenges and resources experienced by the participants in their organizational context. The second session will address the acceptability of wearables and other research tools (e.g., the diary) used in Phase 2b. Informed consent forms will be emailed before the first session and collected in person on the day of the meeting, allowing participants to seek clarification from the researchers if needed. The focus groups will be facilitated by two members of the research team and will be audio-recorded for analysis. At the beginning of Phase 2b, participants will be asked to complete a baseline survey on Qualtrics. The survey will collect demographic, work-related, and health-related information, alongside questionnaires that substantially overlap with those included in the EMA. This will include an electronic informed consent form specific to this phase. Participants must create an anonymous alphanumeric code to associate their EMA responses with physiological data from Fitbit. Upon completing the baseline data collection, two research team members will conduct a debriefing session with all participants at their department. During this meeting, participants will receive a wristband, instructions on its use, and guidelines for completing the EMA questionnaires. Participants will receive instructions in two versions, tailored to their operating system — iOS or Android. The document will contain the following information: a page summarizing the contents of the instructions and researchers’ contact details; a timetable of the monitoring period, indicating the questionnaires to be completed and their active time slots; steps for installing the Fitbit app and configuring the device; steps for installing the Time2rate app and configuring the questionnaires. The EMA questionnaires will be administered using the Time2Rate App, developed by the Bicapp Center (https://www.bicapp.it/it/prodotti/time-2-rate/), which integrates with the Qualtrics system; a ‘tips and recommendations’ paragraph, containing tips (e.g., how to change the Fitbit device’s dominant arm setting) or memoranda for successful monitoring (e.g., remember to keep the Bluetooth on). Each step will be associated with an example screen to facilitate the procedure. Participants will receive assistance installing and using the Time2Rate app on their smartphones, and written instructions containing all relevant information, including researchers’ contact details, will be provided. Participants will be asked to respond to the EMA prompts and wear the Fitbit wristband for 10 consecutive working days. Data collection will be limited to weekdays to reduce participant burden. The EMAs will follow a standard schedule, sending notifications via the Time2Rate app. In the first week, participants are asked to complete an evening EMA focused on the perceived acceptability of the Fitbit. In the second week, when participants are expected to be more accustomed to using the Fitbit, we will collect data on work-related stress and well-being, and perceived acceptability at three different times. We think this could allow us to have information on changing acceptability. The morning EMA will be triggered before the start of the workday between 7.00 and 9.59, with one reminder spaced 60 minutes apart. Following the morning EMA, a daytime EMA will be scheduled at 10.00 to 17.25, and the evening EMA will be prompted after work at 17.30- 23.50, with one reminder sent 2 hours apart. In Phase 2c, semi-structured interviews will be conducted with all participants. These interviews will focus on participants’ experiences with wearing the Fitbit and completing the daily EMA questionnaires. Informed consent will be obtained for audio-recording the interviews, which will be transcribed verbatim and anonymized.

### Instruments and measures

#### Phase 2*a*: Focus groups.

The first focus group (FG1) examines job demands and resources within a specific work context. Participants discuss their well-being at work using photos and post-it notes, identifying both favorable work conditions and stressors. The discussion aims to uncover shared experiences and specific aspects of the work environment. Using an interactive tool (i.e., Mentimeter), participants are invited to answer two questions about job satisfaction and job stress (e.g., “Thinking about your work experience in the last 1 to 10 weeks, how satisfied are you with your job?”). This helps in understanding the work factors linked to this perception. After sharing their definitions of job demands and resources, each participant is asked to list at least one job demand and one job resource. They are also prompted to reflect on how the described situation might be relevant to other colleagues: “To what extent could other colleagues share the situation described?” The second focus group (FG2) investigates the acceptability of a wearable device (Fitbit) and its accompanying app for stress and well-being assessments. Participants engage with the Fitbit, share initial impressions on aesthetics (e.g., “Do you like its design? Would you wear it?”), usability (e.g., “Are you used to using smartwatches?”), and discuss potential applications in the workplace. They examine its benefits (e.g., “What are three positive aspects of using it to assess workplace stress?”) and drawbacks (e.g., “What concerns might arise with daily or nighttime use?”) for stress assessment. Behavioral changes prompted by feedback (e.g., “How would you react to positive or negative data trends?”) are also explored. Participants evaluate the app, sharing their initial reactions, considering their willingness to use a new wellness app, and addressing privacy concerns (e.g., “Would data collection, such as account details, be a concern for you?”). Discussions also cover daily EMA, soliciting feedback on evening or twice-daily entries about significant workplace events and their emotional impact. Finally, participants identify potential barriers for colleagues (e.g., “What privacy or productivity concerns might they have?”) and suggest improvements to the system.

#### Phase 2b: Ecological Momentary Assessment (EMA) and physiological data.

**Fig 2:**
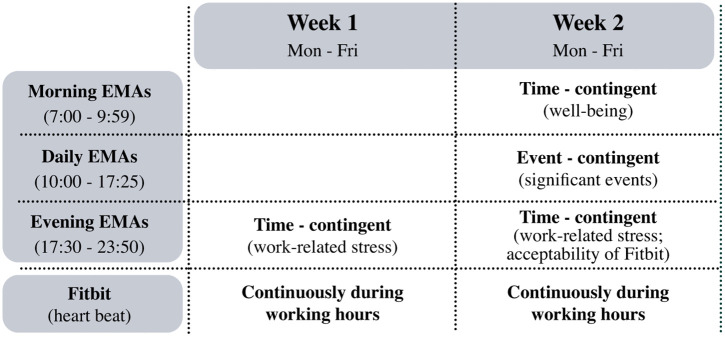
Schedule of EMAs and the wearable device Fitbit during the two weeks.

For data collection, measures were defined for the baseline survey, the first-week EMA, and the second-week EMA, as shown in the schema Fig 2.

**Fig 3:**
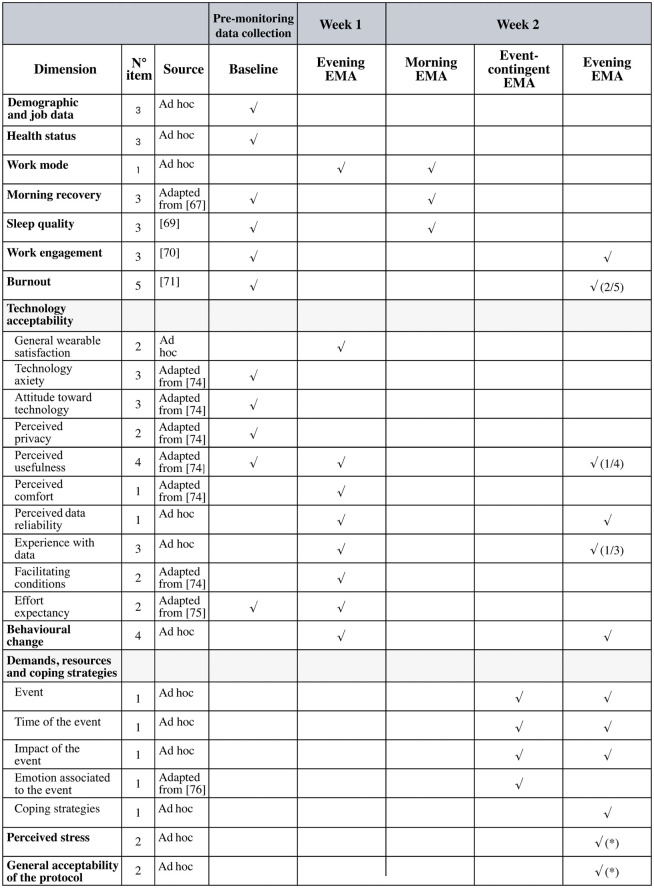
Overview of baseline questionnaire and 2 weeks (ten working days) of EMAs data collection. √ Dimension included in the questionnaire. * Dimension included only the last day of data collection (Friday of week 2).

The baseline questionnaire includes a total of 32 items. The first-week EMA (evening) includes 19 items and has a time-related design. The second-week EMA consists of two time-contingent measurements: morning EMA, 7-item, and evening EMA, 17-item, along with an event-related EMA during work hours. Fig 3 summarizes the dimensions, items, sources, and details of the data collection moments as specified in the paragraphs below.

#### Baseline.

Socio-demographic characteristics such as gender (Please indicate the gender you identify with: male, female, other), role, and age (year of birth) characteristics are measured using single items.

To measure health outcomes in line with the JD-R model, we included

- *Morning Recovery* is a 3-item instrument from Sonnentag and colleagues [66]. The original scale, designed to measure recovery during leisure time, is strongly linked to work engagement and proactivity, and more generally, recovery from work is key for well-being, motivation, and performance [67]. It has demonstrated high internal consistency, with Cronbach’s alphas ranging from.92 to.93. For our study, we adapted the items to specifically measure morning recovery rather than recovery from leisure time, as our focus is on well-being at work. We modified the wording to reflect a person’s feeling of recovery upon waking up, which is a more direct measure of the psychological and physical state relevant to the start of a workday. For example, the original item “Because of the leisure activities pursued yesterday, I feel recovered” was adapted to “In the morning, I feel recovered.” Participants will respond to each item using a 5-point Likert scale, ranging from 1 (totally disagree) to 5 (totally agree).
- *Sleep Quality* is assessed using the 3 items translated from the Minimal Insomnia Symptom Scale (MISS). The original scale, developed by Broman and colleagues [68], assesses core insomnia symptoms. The items are rated on a five-point scale (0 = none, 1 = minor, 2 = moderate, 3 = severe, 4 = very severe). The three items addressed: difficulty falling asleep, night awakenings, and not feeling rested upon waking. Example item: “Please report if you experienced the following problems last night: difficulty falling asleep”. The MISS has demonstrated strong psychometric properties and has been used in various clinical and research settings. In the general population, the scale has shown good internal consistency, with a Cronbach’s alpha of.73.
- *Work Engagement* is measured with the 3 items of the ultra-short measure for work engagement (UWES-3) from Schaufeli [69]. Respondents will answer on a 7-point Likert scale ranging from 0 (never) to 6 (always). The UWES-3 has well-established psychometric properties and has been widely used in occupational health research. A validation study across five countries confirmed its strong internal consistency, with Cronbach’s alpha values ranging from.77 to.85. The scale also demonstrates strong factorial validity, as its single-factor structure is consistently supported, and convergent validity, as it correlates highly with the longer UWES-9 scale and shows expected relationships with other key variables like burnout and job satisfaction. The original UWES items are typically phrased to measure a general, trait-like level of engagement. An example of the item is: “I feel full of energy at my work”.
- *Job Burnout* is assessed by focusing on the emotional exhaustion dimension, using the 5-item Italian validation developed by Borgogni and colleagues [70] of the Maslach Burnout Inventory (MBI) [71]. The MBI is widely considered the “gold standard” instrument for measuring burnout across various occupations and work contexts. The Exhaustion subscale (EE) of the MBI demonstrates strong psychometric qualities and exhibits high internal consistency, with a pooled Cronbach’s alpha of α = .85 across multiple studies. Additionally, it demonstrates significant criterion validity by showing a strong positive relationship with indicators of ill-health, such as mental health disorders/problems (e.g., stress, anxiety, depression). Furthermore, the scale consistently correlates positively with job demands (e.g., workload and emotional demands) and negatively with job resources (e.g., job control), supporting its construct validity within the job demands-resources model [72]. An example item is: “I feel emotionally worn out by my work”. Respondents will answer on a 7-point Likert scale ranging from 0 (never) to 6 (always).

Acceptability will be measured through 12 items adapted from Spagnolli and colleagues’ scale [73]. The original instrument was developed specifically to assess user acceptance of wearable devices in real-world contexts and across different scenarios. Evidence of the instrument’s validity, including Bartlett’s test of sphericity, indicated that correlations between items were sufficient for Principal Component Analysis (PCA). PCA was used to extract 10 components with Eigenvalues over Kaiser’s criterion of 1, explaining 75.53% of the variance. The analysis confirmed the a priori factorial structure of the questionnaire, with most items loading on their intended components, which supports the construct validity. Respondents will answer on a 6-point Likert scale ranging from 1 (completely disagree) to 6 (completely agree). For the present study, we adapted the wording of several items to fit the smartwatch context more precisely, without altering the underlying construct. For example, the original item “I constantly have to deal with information technology” was adapted to “I constantly have to deal with a smartwatch device.” Similar contextual modifications were made across the instrument to ensure relevance, while maintaining the conceptual integrity of the original scale.

Usability will be assessed with 2 items adapted from Lewis [74]. The original PSSUQ is an 18-item questionnaire with a 6-point Likert response scale, validated by Lewis (1992) to have a high overall internal consistency (α ≈ .97) and subscale alphas between.91−.96. It has evidence of validity via factorial analyses, correlations with scenario completion, and with other usability measures. In our adaptation, we have modified the wording of the items to fit the specific context of a Fitbit (for example, the original item “It was simple to use this system” becomes “I think it will be simple to use the Fitbit smartwatch”), while retaining the underlying usability construct. Respondents will answer on a 6-point Likert scale (1 = strongly disagree to 6 = strongly agree).

#### Time-related measures.

The Technology Acceptability of the Fitbit Device is evaluated with a total of 18 items across the two weeks of EMAs. All the items adapted from the previously presented scales were worded to better fit the specific context of Fitbit. For the closed-ended questions, respondents will answer on a 7-point Likert scale ranging from 1 (strongly disagree) to 7 (strongly agree). Likert scale was adapted on a 7-point in order to align the response scale for all the measures across the two weeks of measurements.

- The dimensions of *Technology Anxiety, Attitude toward Technology, Facilitating Conditions, Perceived Usefulness, Perceived Privacy* and *Perceived Comfort* are measured with items adapted from Spagnolli and colleagues [73]. Example of adapted item: “[…]the Fitbit smartwatch device interfered with my work”, starting from the original item “The device would be incompatible with most aspects of my activity (work, sports, research, etc.)”.
- *Ease of Use* is measured with items adapted from Lewis [74]. Starting from the original item: “It was simple to use this system,” an example of an adapted item is “[…] I found it easy to use the Fitbit smartwatch”.

Ad hoc items were created for*:*

- *General Satisfaction*, evaluated with open-ended questions to capture overall experience with the Fitbit for work-related stress assessment. Example item: “Choose an adjective that describes your experience with the Fitbit device today. Briefly explain why you chose this adjective.”
- *Experience with data* to assess participants’ interaction with data, including checking and consulting information on the Fitbit device and app (e.g., “Did you check your Fitbit data (heart rate, steps, etc.) during working hours?”)
- *Perceived Data Reliability*. Example item: “Did the data shown by the device and/or app (heart rate, EDA Scan, sleep) align with your perception of stress?”
- *Behavioral Change* promoted by wearable technologies refers to how these devices influence individuals to modify their actions, habits, or routines to align with health, productivity, or wellness goals. This change is typically driven by continuous monitoring, personalized feedback, and actionable insights. Example item: “Did consulting the data on your Fitbit influence your awareness of your psychophysical state?”.

To measure work-related stress on a day-to-day basis, EMA measures are administered in the morning before work and at the end of the workday. To better align with our daily diary study design, we adapted the items to focus on daily work experiences by introducing wording such as “Today…”. This modification ensures that our measure captures the state-like nature of the investigated dimension on a daily basis, which is essential for assessing within-person changes over time.

Before the workday, outcome measures are assessed: *morning recovery* [66], and *sleep quality* [68].

At the end of the workday, the questionnaire assesses:

- Outcome measures: *work engagement* [69], and *burnout* [70].
- *Job demands and resources*: participants respond to open-ended questions addressing significant work conditions that affected their well-being or distress during the day: “Describe one or two significant events from your workday that affected your well-being”. Participants are instructed to provide a brief description of the stressful or motivating work-related event. No restrictions were imposed on the type or nature of the event (e.g., job-related, relational, organizational), with the only instruction being that it should be an event connected to work-related stress and wellbeing.
- *Coping strategies:* the item of the open-ended question is: “Describe one or two significant events from your workday that affected your well-being” and “Indicate how you dealt with the described situation.”
- The dimensions of “perceived stress” referred to the degree of perceived stress of the workweek will be measured with 2 ad hoc items (e.g., “Thinking about the entire past workweek, overall, how stressful do you consider it to have been?”). Also, the “general acceptability of the protocol” will be measured with 2 ad hoc items (e.g., “If the opportunity arises in the future, I would participate in a data collection on work-related stress as experienced in this study.”). Both dimensions will be investigated only on the last day of data collection (Friday of week 2) and rated on a 7-point Likert scale ranging from 1 (not at all) to 7 (very much).

Where not previously specified, closed-ended questions are rated on an adapted 7-point Likert scale, where 1 indicates ‘strongly disagree’ and 7 indicates ‘strongly agree’. Outcome measures follow the same adaptations as in the baseline questionnaire.

#### Event-contingent.

The event-contingent questionnaire is designed to gather data for assessing daily work-related stress, job demands, and resources. The questions focus on work conditions that are significantly related, according to the JD-R model, to both negative and positive events in workplace well-being. Participants are instructed to complete the questionnaire shortly after an event occurs, providing a brief description of the stressful or motivating work-related event, without prompts from the app. Again, no restrictions were placed on the type of event (e.g., job-related, relational, organizational), as long as it was connected to work-related stress and wellbeing.

For job demands and resources, there is one item: “Indicate the positive or negative event that influenced your workplace well-being.” Participants are then asked when the event took place, its impact on well-being, with an ad hoc closed-ended question, rated on a 7-point response scale (“How did it impact your well-being?”), and the associated emotions, using 8 items from the Laurans & Desmet instrument [75]. The original scale is a non-verbal self-report instrument that uses a set of 14 animated characters to measure distinct categorical emotions (joy, satisfaction, sadness, etc). The instrument’s psychometric properties were established through a series of validation studies conducted across several countries. The studies showed that the animations recognized emotional valence (positive or negative) with up to 95% accuracy. Forced-choice tests also demonstrated 94% accuracy in distinguishing similar emotions. The balance of positive and negative emotions makes the scale highly appropriate for measuring the range of feelings associated with job demands and resources, which typically evoke a mix of positive and negative experiences.

#### Physiological data (wearable device).

Physiological responses will be collected using the Fitbit Charge 5. Fitbit is a commercial off-the-shelf activity tracker consisting of an unobtrusive wristband. This wearable device provides reliable parameters for stress detection, including acceleration, electrodermal activity, heart rate, heart rate variability, and skin temperature. Several previous studies, including a 2020 systematic review by Fuller and colleagues [76], have shown the reliability and validity of these Fitbit-derived measures. For our purposes, we will focus only on heart rate, as it is the only variable measured continuously by the wristband without requiring any additional actions from the users (e.g., activating Bluetooth or enabling smartphone localization). Heart rate is sampled approximately every 5 seconds. Participants will be asked to wear their Fitbit devices and to sync their devices with the Fitbit application on their phones.

#### Phase 2c: Interviews.

At the end of the previous phase, participants will be involved in individual semi-structured interviews [77]. The interviews address the acceptability of the data collection in the monitoring phase. Semi-structured interviews provide detailed information on the experience of participants and are an opportunity for them to express opinions and feelings. The interview will be audio-recorded and subsequently transcribed and anonymized.

#### Phase 3: Data analysis and discussion.

This phase represents the conclusion of the study. Data collected during the previous phases will be compared and analysed by the research team. This phase also involves a discussion about the results with participants. Additionally, the research team will propose potential solutions to enhance work wellbeing.

### Data analysis

The data analysis is expected to provide insights into the average well-being of workers, as well as the demands and resources participants consider relevant in determining workplace well-being. Additionally, the findings are anticipated to highlight the strengths and limitations of using EMA measures and wearable devices for managing and assessing work-related stress and their overall acceptability.

From phase 2. a, the focus group transcripts will be analysed, ensuring the anonymity of participants, through thematic analysis [78]. The analysis is expected to identify the major psychosocial risk factors in the context and their resources. Additionally, valuable evidence is expected to be gathered to explore participants’ perceived usability and acceptance of technology and work-related stress risk evaluation methodology. From phase 2. b, heart rate collected via wearables is expected to correlate with subjective measures obtained from baseline questionnaires and EMA measures. Using a linear mixed model, the analysis will investigate the relationship between heart rate patterns and subjective well-being indicators (burnout, engagement, sleep, recovery, stressor impact). Heart rate data, recorded every 5 seconds (sampling rate ≈ 0.2 Hz), will be synchronized to Italian local time. For visualization and analytic purposes, the time axis will be restricted to 7:00 AM–6:00 PM and resampled to 1-minute intervals. Potential device drift and context-related inaccuracies will be controlled through baseline calibration, within-subject comparisons, and cross-checks on data consistency.

This resampling will allow averaging the heart rate time series and calculating variability ranges for each subject. From the cleaned data, hourly averages and daily mean HR, minimum HR, and median HR values will be computed [79]. HR data will undergo minimum-maximum normalization to standardize values while preserving differences. Artifact correction will exclude implausible heart rate measurements (e.g., HR < 30 bpm or > 220 bpm). Algorithmic detection will identify periods of non-wear or signal loss (e.g., flat lines), which will be removed. Short gaps (≤ 5 minutes) in heart rate signals will be imputed using linear interpolation, while longer gaps (> 30 minutes) will be excluded from primary analyses. To minimize bias, multilevel multiple imputation (MMI) will address within- and between-person variability. Data cleanup will involve removing empty files and duplicated columns. Summary metrics derived from wearable devices will be treated as exploratory; equivalent measures will be recalculated from raw data to validate device summaries. Further, to address Fitbit’s proprietary algorithm limitations, we will focus on heart rate data while monitoring device consistency through baseline checks. Post-assessment checks will be conducted if needed, and potential variability across devices and participants will be accounted for in the analysis using mixed-effects models.

Responses will be scored according to standard procedures (e.g., mean score per scale) for baseline questionnaires and Ecological Momentary Assessment (EMA) items, with reverse-keyed items recoded accordingly. EMA scores will be summarized using a) person-level central tendency (individual mean across EMA occasions) and b) moment-to-moment variability (within-person standard deviation, mean squared successive differences [MSSD]) to capture emotional lability.

We will compare the timing of physiological signals and reported events, the type of events, and participants’ physiological data patterns across individuals. This approach will allow us to explore possible divergences between subjective and objective indicators of stress.

Multilevel mixed models will be employed to handle missing EMA data, with compliance and missingness reported at both participant and daily levels. The HR dataset will be matched with corresponding stressor and stress level data, excluding participants missing either dataset. Content analysis will be employed to categorize daily stressors, while descriptive analysis will identify the most recurrent events within the studied work environment. Further analyses will examine the acceptability of wearable devices and their correlation with the participants’ intention to use them.

The assessment of feasibility and acceptability aims to identify the strengths and weaknesses of the proposed protocol and will be evaluated using both quantitative and qualitative data. These data will then be compared to enhance the robustness of the analysis and provide a more comprehensive understanding of the results. In particular, with the data collected in phase 2.c, the primary expected outcome is an overall assessment of the level of acceptance among participants involved in phase 2.b concerning the data collection methods (Fitbit device and diary). The results of the interviews will be analysed using thematic analysis [80]: this will identify recurring patterns and key themes within the data, providing an understanding of participants’ perspectives. Insights from the interviews will inform strategies for improving the design of future experiments, particularly in the context of data collection aimed at evaluating work-related stress within specific environments. We will also include specific indicators for feasibility and acceptability, such as initial participation rates, adherence, and dropout rates at various stages of the study. Finally, in line with the participatory design principles, the data analysis will also include presenting the results to participants. These results will cover (i) the overall acceptability of the study and (ii) stressors and coping strategies identified within the specific work context. This session serves both as a means of reward for participants’ contributions and as an opportunity to gather feedback on the shared data, ensuring alignment between the findings and participants’ perceptions. Interventions to promote well-being will be discussed collaboratively with participants and the steering committee to assess their feasibility; co-design will be considered as a possible approach for implementing these solutions within the specific work environment.

### Data management and privacy

Privacy concerns have been seriously considered from the early stages of the process. The project has received ethical approval from the Politecnico of Milan Ethics Committee (n° 45/2024) and was evaluated by the local commission for minimal-risk studies of the Psychology Department of the University of Milan-Bicocca (RM-2024–815). The approval was based on the detailed description of the type of data collected, the use and purposes of the collection, and the procedures for data processing (e.g., anonymization, storage place, and duration of data retention). The data treatment and conservation policies comply with the MUSA agreements on collaboration between multiple research stakeholders and co-responsibility. The protocol submitted for approval to the ethics committee includes a detailed data management plan and all the documents employed to guarantee the respect of the rights of the people engaged in the tests, according to the European General Data Protection Regulation (GDPR) and the guidelines for research purposes. The engagement of workers requires the signature of a document on informed consent, with a detailed explanation of the study’s purposes and data controller identification. During phase 2, workers will be asked to use their personal smartphones for data collection. However, participants will log into the applications used in this study (Fitbit and Time2Rate) using a temporary, anonymous account with secret identification codes defined by the users. To link subjective measures from EMA using the time2rate app with Fitbit data, each anonymous account will be associated with an anonymous code generated from the baseline questionnaire by each participant. The connection between the code and the anonymous account will be stored securely, but no personal identifying data will be retained. All data transmission and storage will be encrypted, access will be restricted to authorized research staff, and account-related information will be deleted once the data have been downloaded for analysis. Anonymized transcriptions of focus groups and interviews will be conducted before the analysis. Data management and storage procedures are overseen by this research group, with distinct responsibilities of the experts from the BicApp Laboratory (Bicocca Center for Applied Psychology) and researchers from the IEX-Interaction and Experience Design Lab (Department of Design, Politecnico di Milano). The data management plan and anonymization procedures will be presented and discussed with the participants during the enrolment.

## Discussion

The proposed protocol aims to investigate work-related stress in small and medium-sized workplaces by integrating participatory design principles and methodologies for assessing work-related stress.

A key strength of this study lies in its integration of self-reported data on work-related stressors and health outcomes, collected at multiple time points through focus groups and EMA, with continuous physiological monitoring. This mixed-method approach combines time-contingent and event-contingent sampling, enabling a more comprehensive understanding of routine stress responses and reactions to specific events. Although more complex to code, including open-ended questions about daily events allows for richer qualitative data, minimizing the risk of missing valuable insights. Although a key strength of this study is the integration of physiological and psychological measures in real-world settings—an approach still limited in the literature—we acknowledge that this remains a challenging issue. For this reason, we interpret the two data sources as complementary, with self-reports providing insight into perceived experience and physiological measures capturing underlying bodily responses. Discrepancy may arise because appraisal processes and contextual meaning shape subjective stress ratings, whereas physiological responses reflect autonomic activation that can occur independently of conscious awareness. Previous research has shown that subjective data often do not align with physiological data, due to both biases in self-reports and noise in physiological signals influenced by contextual factors [79]. Nonetheless, examining these measures together in real-world settings represents a valuable step toward understanding how they may complement each other. The study design also incorporates a participatory approach, with discussion sessions to involve participants actively and improve protocol relevance. To address the acceptability of the physiological monitoring device, the protocol integrates insights from both UX Design and Psychology, enabling a better alignment between user comfort and device functionality. Additionally, data on device acceptability are collected over time, with an adaptation period provided before collecting stress data to allow participants to become accustomed to the device. The active involvement of participants throughout the key stages of the process represents the guiding thread in developing this protocol. Data will be shared after each main phase to recognize participants’ contributions and encourage further engagement. A pilot phase involving 10 participants has been completed; full-scale data collection is scheduled to commence next year. Data supporting the findings of this study will be reported in subsequent publications; additional datasets will be made available from the corresponding author upon reasonable request.

### Limitations and future research

A limitation is the short timeframe for monitoring stress, which is limited to 5 days, potentially restricting the assessment of longer-term stress patterns and thus limiting the generalizability of the results. Prior EMA research indicates that even short intensive protocols are sufficient to identify meaningful within-person variability and robust associations between predictors and outcomes [81]. Nevertheless, we acknowledge that the limited monitoring period may not capture cyclical fluctuations in work-related stress that occur over longer time frames, such as monthly deadlines and seasonal workload peaks (e.g., days before the departmental council; during the lecture period), and therefore, the findings should be interpreted as reflecting only the short-term dynamics of workplace stress and recovery. In our study, we aim to provide a realistic and contextually grounded picture of work-related stress by integrating quantitative data with qualitative insights from focus groups and collective discussions.

A methodological consideration concerns the physiological assessment, which relied solely on heart rate data from a commercial Fitbit device. Although this provides a partial view of autonomic stress responses, the use of a low-cost, non-invasive, and user-friendly wearable ensured participant compliance and ecological feasibility in real work settings, where research-grade sensors would have been difficult to implement. Previous studies have shown that Fitbit-derived heart rate data can detect stress-related physiological variations. Nonetheless, heart rate alone cannot capture more specific autonomic indicators, such as heart rate variability or electrodermal activity. Future studies should therefore consider incorporating additional validated physiological measures to enhance accuracy while preserving ecological and economic feasibility.

Another important consideration pertains to the absence of certain complementary measures that could further refine the integration of subjective and physiological data. Although the design of this study permits the combined analysis of perceived and autonomic indicators of work-related stress—an essential strength of this research—we did not incorporate a resting baseline or detailed contextual recordings, such as continuous tracking of physical activity, caffeine consumption, or other behavioral factors that may impact physiological responses. This omission limits our ability to fully disentangle stress-related autonomic changes from other concurrent influences. Consequently, future research should prioritize the inclusion of such baseline and contextual measures, in conjunction with extended monitoring periods, to enhance the ecological validity and interpretative rigor of multimodal stress assessment protocols.

A final limitation of the study is the significant demand placed on participants to regularly respond to Ecological Momentary Assessments (EMAs) and wear a Fitbit. However, ongoing support from researchers—such as assisting with any issues, providing clear instructions, and conducting participative debriefing sessions—could help address this challenge and enhance participant adherence throughout the study.
